# The Meaning of Death: Evolution and Ecology of Apoptosis in Protozoan Parasites

**DOI:** 10.1371/journal.ppat.1002320

**Published:** 2011-12-08

**Authors:** Sarah E. Reece, Laura C. Pollitt, Nick Colegrave, Andy Gardner

**Affiliations:** 1 Institute of Evolutionary Biology, University of Edinburgh, Edinburgh, United Kingdom; 2 Centre for Immunity, Infection and Evolution, Institute of Immunology and Infection Research, University of Edinburgh, Edinburgh, United Kingdom; 3 Department of Zoology, University of Oxford, Oxford, United Kingdom; 4 Balliol College, University of Oxford, Oxford, United Kingdom; University of California San Diego, United States of America

## Abstract

The discovery that an apoptosis-like, programmed cell death (PCD) occurs in a broad range of protozoan parasites offers novel therapeutic tools to treat some of the most serious infectious diseases of humans, companion animals, wildlife, and livestock. Whilst apoptosis is an essential part of normal development, maintenance, and defence in multicellular organisms, its occurrence in unicellular parasites appears counter-intuitive and has proved highly controversial: according to the Darwinian notion of “survival of the fittest”, parasites are expected to evolve strategies to maximise their proliferation, not death. The prevailing, and untested, opinion in the literature is that parasites employ apoptosis to “altruistically” self-regulate the intensity of infection in the host/vector. However, evolutionary theory tells us that at most, this can only be part of the explanation, and other non-mutually exclusive hypotheses must also be tested. Here, we explain the evolutionary concepts that can explain apoptosis in unicellular parasites, highlight the key questions, and outline the approaches required to resolve the controversy over whether parasites “commit suicide”. We highlight the need for integration of proximate and functional approaches into an evolutionary framework to understand apoptosis in unicellular parasites. Understanding how, when, and why parasites employ apoptosis is central to targeting this process with interventions that are sustainable in the face of parasite evolution.

## Introduction

Cell death programs, such as apoptosis, play essential and well-documented roles in the development and maintenance of multicellular organisms [1]. The evolution of programmed cell death (PCD) in multicellular organisms is readily explained because an individual's cells are clonally related and so have a shared goal in the successful development and maintenance of the organism [2], [3]. For this reason, cell death by genetically controlled and tightly regulated processes was assumed only to have evolved in multicellular taxa [4], [5]. However, there is mounting evidence that forms of apoptosis occur in unicellular protozoan parasites, but whether this is apoptosis has proved controversial and also has stimulated much debate about the evolutionary origins of PCD [6]–[13].

The existence of apoptosis mechanisms in protozoan parasites offers the potential to subvert them and develop novel therapeutic tools for some of the most serious infectious diseases of humans, companion animals, wildlife, and livestock. Whilst research into the mechanisms involved in parasite apoptosis is progressing rapidly, the evolutionary understanding for why apoptosis occurs in parasites is at best speculative, and at worst misleading. Yet, an integrated understanding of how, when, and why parasites employ apoptosis is central to targeting apoptosis with interventions that are sustainable in the face of rapid parasite evolution. Here, we set up the central evolutionary concepts that are expected to ultimately explain apoptosis in these organisms and outline the key hypotheses to test and the approaches required. We focus on malaria (*Plasmodium*) parasites, but the evolutionary principles apply more broadly to other parasite taxa.

## Programmed Cell Death Processes

**Figure 1:**
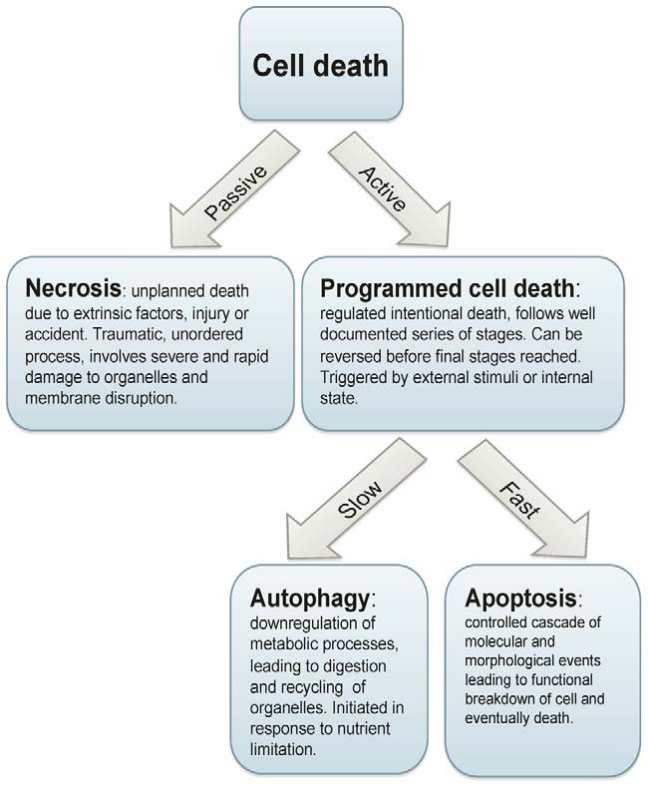
Cell death processes.

PCD in any organism is characterised by a cascade of controlled events that eventually become irreversible and lead to cell death (Figure 1). In multicellular organisms, autophagy and apoptosis are recognised as the two main types of genetically encoded processes leading to cell death. Broadly, autophagy (*self digestion*) is thought to be a process that cells at risk of starvation can undergo to maximise their chances of surviving until conditions improve. Autophagy also plays a key role in the cellular re-organisation required during developmental transitions. Thus, in most cases, autophagy may best be viewed as a survival strategy for *avoiding* death. In contrast, apoptosis is a genetically regulated execution process that leads *directly* to death. In multicellular organisms, apoptosis is essential for proper development, homeostasis, and the immune response; for example, cells are disassembled and cleared without causing harmful inflammation [14], [15]. Necrosis is usually considered a third type of death and encompasses processes that occur during accidental cell death.

Apoptosis of cells in multicellular animals is diagnosed when some, or all, of the following morphological (phenotypic) characteristics are observed: DNA fragmentation, chromatin condensation, membrane blebbing, formation of apoptotic bodies, cell shrinking, translocation of phosphatidylserine to the outside of the plasma membrane, cleavage of proteins by caspases, loss of membrane potential, and release of proteins from mitochondria [16]–[18]. Necrotic death does not normally involve these markers because severe damage resulting in accidental death generally causes rapid membrane permeability and leakage of cell contents. A range of apoptosis markers have recently been observed in a diverse range of unicellular taxa, including bacteria, *Blastocystis, Chlammydomonas, Dictyostelium, Giardia, Leishmania, Plasmodium, Saccharomyces, Tetrahymena, Trichomnas,* and *Trypanosoma* [19]–[28].

## Programmed Cell Death in *Plasmodium*?

**Figure 2:**
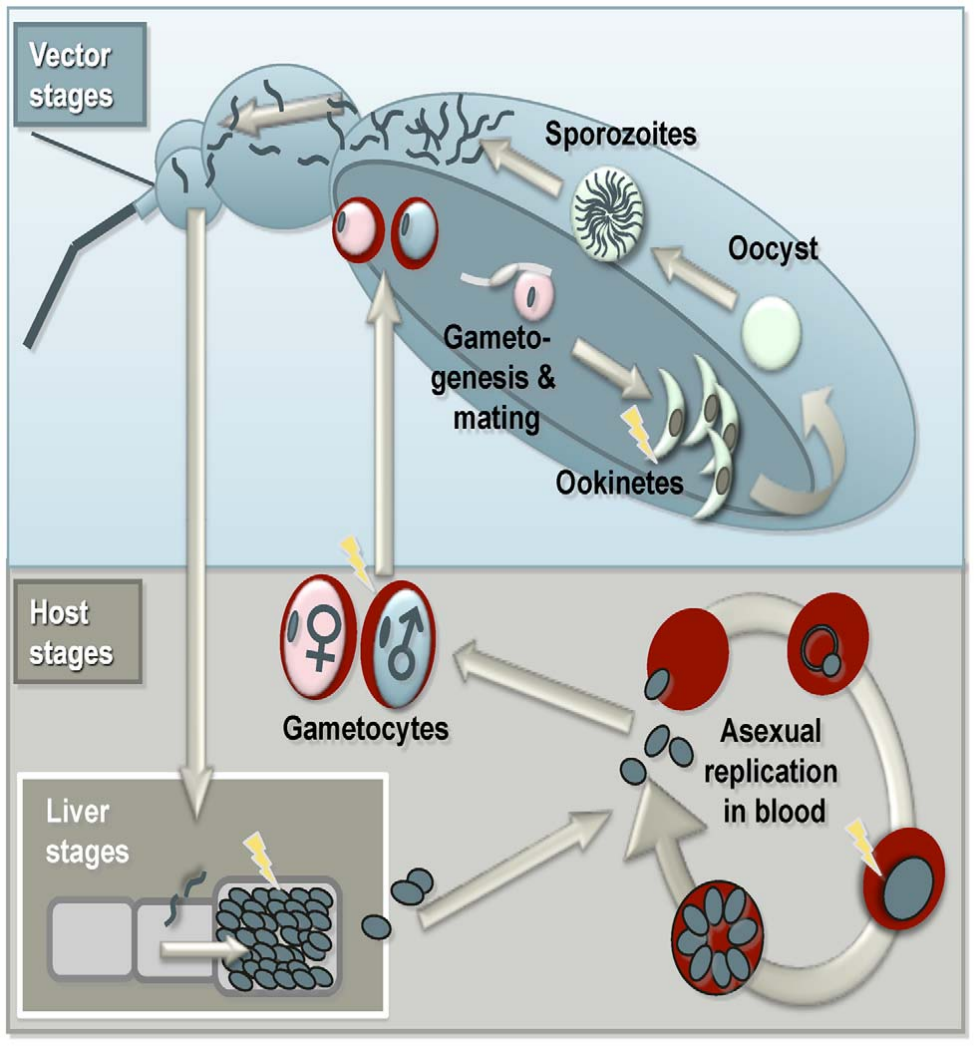
Summary of the malaria life cycle and apoptosis. Stages in which apoptosis has been observed are highlighted with lightning bolts.

Multiple markers of apotosis have been observed in stages of the *Plasmodium* life cycle (Figure 2) that occur in the vector (ookinetes) and the host (liver schizonts and blood stages) [21], [25], [26], [29]–[34]. Most studies to date have focussed on the rodent malaria parasite *Plasmodium berghei,* and the markers that have been observed include chromatin condensation, DNA fragmentation, externalisation of phosphatidylserine, apoptotic bodies, and the activity of caspase-like proteases [21], [29], [30]. Results from studies focussing on the human parasite *P. falciparum* are mixed but report apoptosis-like death in mosquito (ookinete) stages [29], and vertebrate blood (asexual and gametocyte [26], [33], [34]) and liver stages [31].

Despite the number of studies reporting these markers in a diverse range of unicellular organisms, the occurrence of apoptosis in protozoan parasites has proved controversial [12], [35]. The problems span from contradictory data to a lack of consensus on the evolutionary explanations and the mechanisms involved [7], [16], [30], [36]. The evolutionary explanations are the focus of this review, and we explain how progress on this front could reconcile the apparently contradictory data. The framework we propose integrates evolutionary biology, ecology, cell and molecular biology, and host–parasite interactions. A central concept is that we distinguish between the evolution of the *proximate mechanisms* that enable a cell to execute itself and the *ultimate explanations* for why a cell should execute itself (i.e., the *how* and *why* questions). In the following sections we introduce the evolutionary conundrums involved in parasite apoptosis, explain how natural selection solves these problems, and outline the studies required to move the field forward.

## Ultimate Explanations: When and Why Is Suicide Adaptive?

**Figure 3:**
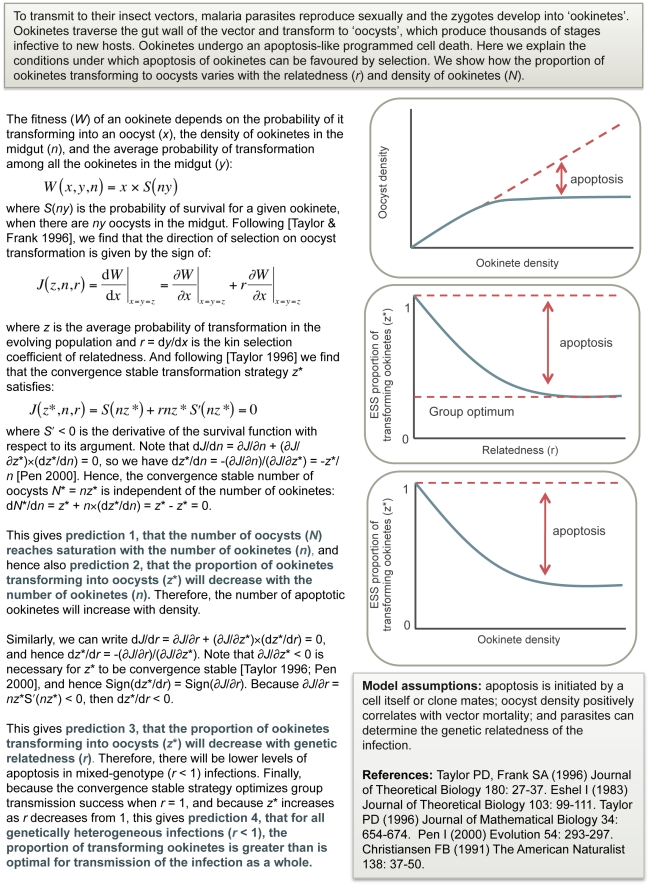
Evolutionary theory for altruistic suicide. Using *Plasmodium* ookinetes as a case study, we describe the scenarios under which natural selection would favour the evolution of an apoptosis strategy and illustrate how rates of apoptosis are predicted to vary.

The occurrence of apoptosis in unicellular taxa presents a challenge for evolutionary theory [37]–[39]. The central issues are explained below and illustrated in Figure 3 for the case of ookinetes. Intuitively, the Darwinian notion of “survival of the fittest” suggests that parasites should evolve strategies to maximise their proliferation, not their death. The evolution of altruistic behaviours poses a major evolutionary conundrum: why should an individual, or cell, do something to benefit others at a cost to its personal reproductive success [2], [3], [40], [41]? In the evolutionary literature, explanations for altruism behaviours had a rocky start due to group adaptationist arguments, which claimed individuals cooperate for the good of the group [2], [42]. Despite being overturned several decades ago, group adaptationism persists and manifests most often as statements about cooperation facilitating the survival of the species or population. By explaining the modern evolutionary framework, and the relevant semantics (Table 1), we hope to avoid another discipline having to repeat this debate. The key point is that natural selection leads to adaptation of individuals. In exceptional circumstances, natural selection can also drive the adaptation of groups, but competition between individuals usually destroys the common good that comes from cooperating. This concept can be illustrated by considering common land available for grazing to the livestock of many herdsmen [43], [44]. If a herdsman receives earnings only from his animals, the best strategy will be to extract as much benefit from the pasture as possible, adding more animals than other herdsmen even if the land becomes barren as a result. This so-called tragedy of the commons arises because the benefit from each animal goes directly to its owner, whilst all herdsmen share the cost of reducing the quality of the land. Cooperation can also be eroded by the evolution of cheating strategies [45]. In our example, overall productivity is maximised if herdsmen cooperate to maintain the land while sharing the profits, and the tragedy of the commons looks like it can be avoided. However, now the best strategy for a herdsman is to cheat by taking his share of the profits without contributing as much work as the others.

**Table 1:** Social semantics.

**Actor**	The individual who performs the behaviour or expresses the trait of interest.
**Altruism**	A behaviour or trait that is maintained by natural selection because other individuals benefit when the behaviour is performed or the trait is expressed—*at a cost to the actor*.
**Clone**	Genetically identical parasites. For example, a single malaria parasite is capable of establishing an infection through asexual replication (clonal expansion) and these parasites will be genetically identical clone-mates.
**Cooperation**	A behaviour or trait that is maintained by natural selection because other individuals benefit when the behaviour is performed or the trait is expressed.
**Fitness**	Success in transmitting one's genes into future generations, which for parasites will usually be the number of new hosts. Fitness can be accrued *directly*, through transmission of a genotype's own genes, or *indirectly*, through alleles shared with related parasite genotypes.
**Genotype**	See clone.
**Inbreeding**	The probability of genetically similar parasites recombining (mating).
**Infection**	The parasites sharing a host. May all be genetically identical to one another or composed of a mixture of co-infecting parasite genotypes and/or species.
**Killing**	When a parasite cell is induced or coerced into undergoing apoptosis by unrelated parasites or host/vector immune responses.
**Kin selection**	The component of natural selection that maintains a behaviour or trait in a population because relatives benefit when the behaviour is performed or the trait is expressed.
**Relatedness**	Genetic similarity between individuals. The proportion of alleles that are identical by descent between individuals. It is important to note that although cohorts of asexually replicating parasites will vary in the genes being expressed over the course of a single infection, they are isogenic to the original infecting parasite clone.
**Recipient**	The individual that is affected (positively or negatively) by the Actor's behaviour or traits.
**Suicide**	Intended death executed by the individual parasite cell (Actor). Incurs the largest cost to personal fitness.

## Relatedness Regulates Death

Given that competition and cheating can prevent cooperation, under what circumstances can altruistic behaviour evolve and be maintained? The solution is to notice that natural selection adapts individuals to transmit copies of their genes to future generations, and they can do this either by promoting their own reproductive success (*direct fitness*) or else by promoting the reproductive success of their relatives, who tend to share copies of the same genes (*indirect fitness*). Consequently, individuals are adapted to maximise the sum of their direct and indirect fitness—their *inclusive fitness*—rather than their personal fitness [3], [40], [46]. Thus, the more closely related the interacting individuals are, the bigger the fitness payoff from helping each other to reproduce. Specifically, cooperation can evolve when the benefit (*b*) provided to a recipient, weighted by the relatedness (*r*) between the recipient and actor, is greater than the cost (*c*), paid by the actor through altruism (i.e., when Hamilton's rule *rb–c>*0 is satisfied) [3], [40]. The coefficient of relatedness (*r*) is a regression measure of genetic similarity between two individuals, relative to the average similarity of all interacting individuals [3], [47], [48]. Notably, relatedness is unity (*r* = 1) in single-clone infections so, in this special case, altruism is favoured when the benefit to the recipient exceeds the cost to the altruist (*b*>*c*), and selection acts according to what's best for the group [2], [3].

Hamilton's rule tells us that for actors to pay a big cost of helping (e.g., suicide), the interacting individuals must be closely related and recipients must get a considerable benefit. Crucially, cells within a metazoan individual are clonally related (genetically identical), and so have identical evolutionary interests. Thus, apoptosis in metazoans is easy to understand—clearly, an organism can only survive and reproduce as long as its many differentiated and integrated cell types cooperate to maintain the body as whole. Do the same rules apply to parasites? Different specialised forms are responsible for in-host replication and between-host transmission, and infections are established and maintained through clonal expansion. Evolutionary theory predicts that an infection of clonally related parasites will have a shared goal of optimising transmission to mosquitoes and thus should behave in ways analogous to multicellular organisms, i.e., as a single fitness-maximising individual [2], [49], [50]. Therefore, if apoptosis is a cooperative trait, it will be more frequent in infections in which parasites are genetically related, to ensure that those undergoing apoptosis provide a benefit to their kin. In genetically diverse infections, parasites have no fitness interest in paying the cost of apoptosis to benefit non-kin; undergoing apoptosis in a mixed infection may therefore represent a serious error, because competitors will benefit from the sacrifice. Evolutionary theory predicts that how many parasites undergo apoptosis will depend on what is optimal for *each clone* in an infection, not what is optimal for the *infection* as a whole. This means that the level of apoptosis required to maximally benefit each surviving recipient will only occur in clonal infections, and parasites in mixed infections face a tragedy of the commons.

### Density-Dependent Death

Identifying who benefits from apoptosis is important, but does not ultimately explain why a parasite should help others at a cost to itself—and self-destruction exacts the ultimate price—as the nature of the benefits must also be identified. Under what circumstances would reducing parasite number provide assistance to the survivors? If host or vector survival is negatively related to infection intensity, apoptosis could prevent premature death of hosts or vectors [9], [51]. This could explain why huge bottlenecks occur between developmental stages in mosquitoes and resolve the issue of whether malaria is harmful to mosquitoes (L. Pollitt, T. Churcher, E. Dawes, N. Colegrave, S. Reece, et al., unpublished data). Apoptosis has also been proposed to act as a form of selection (by removing suboptimal or damaged parasites, only the best can propagate) [52]; as a mechanism to avoid limitation of nutrients and resources as a result of crowding [30]; or because high numbers of ookinetes crossing the midgut may activate mosquito immune responses that damage parasites [53]. None of these explanations are mutually exclusive and could act together to make apoptosis worthwhile.

If apoptosis is used to regulate numbers, the benefits to survivors must depend on their density. If parasites are at low density and host/vector survival is not at risk, there is no benefit from any parasites undergoing apoptosis. Moreover, parasites at low density are vulnerable to clearance by the host/vector immune system, so parasites undergoing apoptosis would increase this risk. But if parasites are at a density where host/vector survival is at risk, the best strategy is for enough parasites to undergo apoptosis to maintain a sub-lethal density. The same logic applies if apoptosis is used to regulate competition between parasites for resources, or to avoid activating mosquito immune responses: apoptosis is only beneficial when parasites are at a sufficiently high density that resources become limiting or the immune system activates. For all of these scenarios, the occurrence of apoptosis should depend on parasite density—the more parasites there are, the bigger the benefits to survivors—so the proportion of parasites undergoing apoptosis increases with their density.

### Sophisticated Strategies?

How could parasites detect information about relatedness and density and determine if their circumstances merit apoptosis or proliferation? In fact, it is not necessary to actively detect this information, as natural selection can shape parasite strategies in line with the average densities and relatedness of parasites encountered in infections. For instance, constitutively high levels of apoptosis may evolve in populations where infections are largely clonal (for example, in areas with low transmission), whereas apoptosis will occur at lower rates in areas where mixed infections are the norm. However, a “one-size-fits-all” strategy is a poor solution when variation in parasite density and relatedness is experienced during infections and in different hosts. If parasites are able to detect information about the relatedness and density of their infection, more complex strategies become possible. An obvious parallel here is the regulation of social behaviours in bacteria through quorum sensing [54]. For example, the proportion of parasites within a clone that undergo apoptosis may be plastic and facultatively adjusted in response to variation in the presence of co-infecting clones and the density of kin and non-kin. In mixed infections, the abundance of co-infecting clones varies, and so parasites of a rare clone have much less to gain from undergoing apoptosis, as their sacrifice will disproportionately benefit parasites of the dominant clone.

Such finely tuned strategies may seem unrealistic, but recent work has demonstrated that parasites can determine the genetic diversity of their infections and also suggests that they measure the density or proliferation rate of clone-mates [55]–[59]. Perhaps the stress factors known to elicit apoptosis are actually detected by parasites to gather information about density and relatedness. Interestingly, genetic kin discrimination mechanisms are rarely found in nature, but host–parasite interactions are a scenario where they can evolve [60]–[62]. The destruction of mosquito epithelial cells caused by ookinetes penetrating the midgut could result in the release of factors that provide information about density, and once these factors reach a threshold level, the remaining ookinetes undergo apoptosis. The expression of apoptosis also has to be probabilistic or all parasites carrying the genes for apoptosis will die (i.e., a constitutively expressed suicide trait cannot evolve). This could be achieved by a conditional expression mechanism, for example, only parasites with a poor internal state may undergo apoptosis, or the spatial arrangement of parasites in host compartments may be such that only those in very high local densities undergo apoptosis. Such local spatial structuring enables the probabilistic expression of cooperative suicide in *Salmonella typhi* [63].

## Disentangling Suicide from Other Deaths

So far, the literature has focussed on the view that apoptosis is “altruistic suicide” if it is an active process that parasites undergo to benefit their relatives. However, there are further complications with the connotations of “suicide”, and alternative explanations must be considered.

### Death as a Default

Uncovering the explanations for why parasites die does not necessarily explain why they do so by undergoing apoptosis—why use an energy-demanding process to die rather than simply terminating development and eventually dying by necrosis? There are several non-mutually exclusive explanations. To intentionally and permanently halt development/metabolism, an active process may be required. If blood stage parasites—particularly female gametocytes—have the machinery to undergo apoptosis, then ookinetes could co-opt these mechanisms in order to bring about their own death. In mammalian cells, apoptosis appears to be the “default” and death needs to be constantly repressed [5]. This situation could ensure that when anything goes wrong with a cell it dies, and so risk to the organism is minimised.

In some cases, apoptosis might result in the release of cell contents that are beneficial to others [64], and it might be advantageous for damaged or senescing blood stage parasites to die in a way that minimises the exposure of their antigens to the host immune system (so parasites may use phosphatidylserine to manipulate phagocytes into tidying them away). Using apoptosis to die could be particularly important for senescent gametocytes, as it would minimise the development of transmission-blocking immune responses to their antigens, and could explain why metacaspase 1 is expressed in sexual but not asexual blood stages. The ultimate explanation for apoptosis in this case is still kin selection, as apoptosis enables dying parasites to maximise the transmission of future clone-mates. However, the costs of apoptosis are minimised if parasites are going to die anyway (e.g., due to senescence).

Alternatively, apoptosis in parasites could be an unfortunate—but unavoidable—consequence of natural selection acting on other cellular processes or traits [37]. Natural selection would not remove such a deleterious trait from the population if the genes involved prove sufficiently beneficial to other processes. In support of this idea, metacaspase genes in algae are implicated in housekeeping processes [65]. Experiments that interfere with the expression of apoptosis to quantify fitness consequences or phenotypic changes must be careful to account for correlated effects on other traits linked by pleiotropy. It is also possible that apoptosis could have initially occurred as a result of pleiotropic effects, but thereafter been maintained owing to the benefits to kin, and is thus shaped by selection as an altruistic trait (for example, ookinetes that develop from older female gametocytes may be more likely to undergo apoptosis to benefit others). This scenario highlights the potential for differences between the selection pressures that explain the origin and maintenance of traits.

### Coercion and Killing

From an evolutionary perspective, there is a big difference between apoptosis arising from a cell making its own decision to die versus it being coerced into doing so by others [66]. Whilst phenomenologically these alternatives seem the same, there is an important distinction for understanding how natural selection operates on these traits [67]. Coercion could be a result of host/vector responses to infection or a result of interactions between unrelated parasites. If apoptosis mechanisms can be activated by a cell's own state and/or in response to information it detects about the environment, a cell can make its own decision to die. If other cells can hijack this system and directly induce apoptosis in others, or provide misinformation to manipulate others' perception of density and relatedness, this is coercion. In clonal groups, suicide and coercion amount to the same thing because the fitness interests of all co-infecting parasites are aligned [2], [3], but the situation becomes more complex in mixed infections as parasites may be killing non-kin to benefit themselves and their relatives. This scenario still relies on there being benefits of reducing parasite number, but now predicts that apoptosis will be positively correlated with the genetic diversity of the infection. Given that apoptosis has been observed in clonal infections, a warfare explanation seems unlikely, but the relationship between levels of apoptosis and relatedness will unambiguously reveal whether cooperation or conflict is the best explanation.

Another type of coercion occurs if host/vector factors induce parasites to undergo apoptosis, resulting in an elaborate way to be killed. For example, reactive oxygen species and derivatives can induce apoptosis in ookinetes (but this is not the only cue, as apoptosis occurs *in vitro*) in the absence of influences from both host and vector [21], [32], [68]). These studies highlight the likely complex nature of apoptosis and suggest that it may be driven by a combination of parasite strategies and host/vector responses to infection. Unfortunately, the influence of host/vector factors will make it more complicated to undertake definitive tests of the cooperation/conflict explanations for apoptosis, but the stronger the influence of immune factors, the weaker the relationships between apoptosis, parasite density, and relatedness will be.

## Proximate Mechanisms: From Mammals to Malaria

Whilst the literature on mammalian cell death undoubtedly has many valuable lessons for parasitologists, there is still confusion about how many distinct types of PCD exist, let alone how the underlying processes should be defined and detected [16]. Given the complexity of apoptosis in multicellular taxa and the lively debate surrounding these issues, we propose that focussing on the mechanistic differences between parasite and metazoan apoptosis without the relevant ecological context is not a useful way to progress. Furthermore, given that parasite apoptosis mechanisms are likely to be ancestral to processes in multicellular organisms, using mammalian apoptosis as a template may lead to overcomplicated expectations for what should be observed in parasites and so create confusion over the naming of processes observed in parasites. Below, we illustrate two examples where the use of apoptosis markers developed for mammalian cells are challenged by a parasite-centred perspective.

### Tidy or Untidy Death?

The activities of caspase-like molecules and DNA fragmentation are the most often observed markers of apoptosis in parasites, whereas the formation of apoptotic bodies and phosphatidylserine externalisation have been documented, but less often [21]. These are key markers of apoptosis in mammalian cells, so why do they seem to be uncommon in parasites? In mammals, apoptotic bodies package up cellular contents, and phosphatidylserine provides “eat me” signals to phagocytes and macrophages to engulf the bodies. The engulfment of apoptotic bodies by phagocytes prevents cellular contents being exposed to the immune system and modulates responses by suppressing inflammation, modulating cell killing, and regulating immune responses [17]. Whilst this is clearly important for mammalian homeostasis, it is not obvious that such a tidy death should be a concern for ookinetes in a mosquito midgut (and phagocytes in a blood meal are unlikely to be functional for long).

### Caspase-Independent Execution?

In multicellular taxa, apoptosis is by members of the caspase family of clan CD cysteine proteases [69]. Whilst assays developed for mammalian caspases detect activity in ookinetes [21], [30], there are no homologues of caspase genes in the *Plasmodium* genome [70]. Three metacaspse genes have been identified, which are also clan CD proteases and may be distantly related to ancient caspase families [12], [71]. Of these, *Plasmodium* metacaspase 1 (PxMC1) is expressed in female gametocytes and all mosquito stages but not in asexual blood stages. In one study [7], disruption of this gene in *P. berghei* did not result in an apoptosis rescue phenotype. However, in that study, the numbers of parasites exhibiting apoptosis markers in the wild-type control was lower than has been reported elsewhere. More recent work also suggests that PbMC1 is not essential for the execution of an apoptosis programme, but may be involved in its initiation (L. Pollitt, T. Churcher, E. Dawes, N. Colegrave, S. Reece, et al., unpublished data). In contrast, evidence supporting a caspase-like executioner comes from identification of a putative tudor staphylococcal nuclease (TSN)-like substrate [36], *in vivo* data showing that a variety of general and specific caspase inhibitors prevent ookinete death [21], and the involvement of clan CA cysteine proteases in chloroquine-mediated apoptosis [33]. However, these inhibitors may have off-target effects, and the picture is made more complex by the recent consensus that apoptosis can occur through caspase-independent mechanisms [72], [73]. The questions of whether metacaspases are essential or involved at all in apoptosis, what caspase assays actually detect in parasites, and whether this target is involved in apoptosis, all remain to be answered. Work on the molecular and cellular mechanisms involved in the initiation and orchestration of apoptosis has progressed further for *Leishmania* and *Trypanosoma* than *Plasmodium.*

## Future Directions

Current thinking proposes that apoptosis is an altruistic trait used to regulate parasite numbers and prolong host/vector survival. Testing the relationships between apoptosis, density, and the genetic diversity of infections is key to resolving this (Table 2). Parasites could also benefit from inducing their relatives to undergo apoptosis if those that are sacrificed are inferior: for example, senescent gametocytes may be more likely to undergo apoptosis than infectious ones. Methods to isolate and compare the genetic and phenotypic qualities of parasites exhibiting apoptosis markers to those continuing development are needed. A re-evaluation of how apoptosis is detected is required (Table 2), as assays should also be undertaken with reference to when apoptosis is likely to be initiated and the resulting markers have had the time required to develop. To achieve this, a better understanding of the mechanisms involved in apoptosis is clearly required.

**Table 2:** Key challenges and outstanding questions.

**1. Are there density-dependent benefits?** In general, it is very difficult to quantify the fitness consequences of variation in a trait. The clearest results would come from experiments where parasite apoptosis (or response to density) is prevented and the resulting transmission compared to that of parasites able to undergo apoptosis (or respond to density). Alternatively, some progress can be made if studies reporting the proportion of parasites displaying apoptosis markers did so in relation to the number in the sample examined.
**2. Do related or unrelated parasites, or hosts, or vectors benefit?** Experiments of the type described above in which parasites are “tricked” or prevented from undergoing apoptosis are also required to evaluate who benefits. Benefits for parasites can be diverse, from preserving the host/vector, to reducing competition for resources, to controlling the development of immune responses. These benefits may not be mutually exclusive, and in the case of preserving the host/vector, the parasites and the host/vector will both benefit.
**3. What are the mechanisms involved?** Understanding how apoptosis is initiated and executed is central to interfering with it in a therapeutic context, but is also required to determine if apoptosis is adaptive or a by-product of other processes. For example, apoptosis may have evolved to be initiated in certain circumstances by some life cycle stages, but the machinery may be constrained and thus is vulnerable to activation by environmental factors in the host/vector (e.g., oxidative stress), which may be detrimental in different life cycle stages.
**4. What are the signal/cues involved?** If apoptosis varies with density and relatedness, how is this information detected? Experiments suggest that blood stage *Plasmodium* adjust the production and sex ratio of gametocytes in response to density and relatedness. Do female gametocytes retain this information for use as ookinetes, or do ookinetes gather their own information? Given the variation in activity of transmission-blocking immune factors in the blood meal, parasite density in the host may not correlate closely to density in the vector.
**5. Which markers should be applied and when?** Assays should be applied that match the schedule of decision-making life cycle stages with time taken to progress far enough into the apoptosis program to display the morphology being assayed. There is a potential trade-off here: detecting apoptosis later in the program is useful as it is less likely to be reversed, but there is a risk of assaying too late and detecting only the resulting necrosis. Experiments to verify that parasites with apoptosis morphologies do actually die are also needed. It may be possible to sort healthy and apoptotic ookinetes to test if only healthy parasites transform into oocysts.
**6. What is the evolutionary potential of PCD?** Genetic variation for the expression of a trait is the raw material for natural selection. To predict the evolutionary response of targeting apoptosis with an intervention, data on levels of genetic variation for rates of apoptosis under a range of density and relatedness conditions are needed. In particular, identifying whether parasites have fixed or facultative strategies for undergoing apoptosis is key, as a plastic apoptosis strategy may either facilitate or constrain the evolution of fixed strategies. Integrating this information with an understanding of the mechanistic constraints on apoptosis would provide a solid base for forecasting parasite evolution.
**7. Who dies?** If an apoptosis strategy is adaptive it must be managed as a trait with probabilistic expression (a trait cannot evolve if it kills all individuals). How is this ensured? Does compartmentalisation of parasites in the host/vector generate variation in density information? If so, how accurate is this information? Are only a proportion of parasites responsive to the density and relatedness conditions favouring apoptosis? Perhaps only parasites with inferior phenotypes (senescent) undergo apoptosis? To what extent is execution initiated in response to intracellular state and “signals” from other parasites in the host/vector?

The underlying genes and mechanisms orchestrating these morphologies in protozoan parasites appear to differ from those in multicellular taxa, offering an opportunity for novel treatment strategies. The value of an evolutionary framework to evaluate and predict the long- and short-term success of interventions is increasingly being recognised. Consequently, understanding when and why parasites undergo apoptosis is as important as understanding how they do this. The fastest progress is likely to be made by integrating evolutionary and functional methodologies, but the success of this approach hinges on effective communication between fields. Misconceptions and semantic distractions can easily pervade the literature of new fields and hinder progress. Putting apoptosis in protozoan parasites into a coherent evolutionary framework whilst the topic is in its infancy facilitates progress in this field.
